# The Theory and Practice of Active Aging

**DOI:** 10.1155/2012/420637

**Published:** 2012-10-21

**Authors:** James F. Fries

**Affiliations:** Department of Medicine, Stanford University School of Medicine, 1000 Welch Road, Suite 203, Palo Alto, CA 94304, USA

## Abstract

“Active aging” connotes a radically nontraditional paradigm of aging which posits possible improvement in health despite increasing longevity. The new paradigm is based upon postponing functional declines more than mortality declines and compressing morbidity into a shorter period later in life. This paradigm (Compression of Morbidity) contrasts with the old, where increasing longevity inevitably leads to increasing morbidity. We have focused our research on controlled longitudinal studies of aging. The Runners and Community Controls study began at age 58 in 1984 and the Health Risk Cohorts study at age 70 in 1986. We noted that disability was postponed by 14 to 16 years in vigorous exercisers compared with controls and postponed by 10 years in low-risk cohorts compared with higher risk. Mortality was also postponed, but too few persons had died for valid comparison of mortality and morbidity. With the new data presented here, age at death at 30% mortality is postponed by 7 years in Runners and age at death at 50% (median) mortality by 3.3 years compared to controls. Postponement of disability is more than double that of mortality in both studies. These differences increase over time, occur in all subgroups, and persist after statistical adjustment.

## 1. Introduction

“Active aging” and the related terms “healthy aging,” “successful aging,” “productive aging,” “aging well,” “living well,” “senior wellness,” and “compression of morbidity” endorse a radically nontraditional paradigm of human aging, which includes gains as well as losses and which posits possible improvement in future human health despite increasing longevity. Each of these terms, discussed briefly below, foresees a new paradigm for gerontology, based upon postponing functional declines into older ages with a goal of postponement of morbidity more than mortality, compressing morbidity into a shorter period later in life, and decreasing cumulative lifetime morbidity [1]. The new paradigm contrasts strikingly with the old “Failures of Success” paradigm, where improvements in longevity would inevitably lead to ever larger numbers of persons in ever poorer health [2]. 

There are differences in nuance between these terms and in the metrics by which they might be measured, and confusion might be reduced by greater agreement on terminology. We are most comfortable with “Compression of Morbidity” since it implies a strategy for improving health, the theory behind the strategy, and the means of testing progress, albeit a more technical term than alternatives. Of alternative terms, we prefer the term “healthy aging” since it includes the notion of improving each of physical health, mental health, and social health, whereas “active” seems more focused on the physical component of health, “productive” on some form of work product, “successful” on a quite narrow definition of aging, and “well” on the absence of disease. None of these terms are universally endorsed, but we need to recognize the common themes in these various restatements of the new paradigm.

Morbidity, in common usage, is a general term for the absence of health, and disability is the most frequently used metric for estimation of morbidity. Morbidity itself is an imprecise term often defined in different ways, usually denoting impaired health of some kind other than death. Morbidity itself does not have an agreed metric for its study. In practice, the most frequently used metric for estimation of morbidity has been ability at activities of daily living (ADL) as measured by the Health Assessment Questionnaire (HAQ) Disability Index [3] or similar instruments [4]. Such instruments measure physical capacity and disability on a continuous scale and indirectly include the cognitive abilities which ultimately direct the physical activity and the social environment which enables it [5]. 

This paper attempts to pull together evolving theory and evolving practice, with an emphasis on the history of the compression paradigm and the presentation of new longitudinal data over a twenty-year period now confirming compression of morbidity by lifestyle choices under certain conditions.

## 2. Theory

Over thirty years ago when I first began to examine the postulates of gerontology and human aging I did so from a background in medicine, rheumatology, clinical epidemiology, health outcomes research, and health policy, with an emphasis on prevention and on outcomes of chronic illness [6]. From this perspective, it seemed clear that much diminished capacity could be postponed or even prevented at the individual level and thus potentially at the population level. It also seemed clear that overall national improvements in health would likely require reduction in health risks [7, 8]. Thus, the marked reduction in heart disease mortality beginning in the nineteen-sixties was associated with decreases in risk factors such as smoking and cholesterol levels. These clearly affected both age-specific incidence rates and mortality rates. Disease-associated morbidity from heart disease now developed later in life and mortality also was postponed. An evolving challenge was to develop risk factor models on a population basis rather than a disease-specific one, since allocation of disability and other outcomes to specific diseases is difficult and competing risks make apportionment inaccurate as well.

There is a clear dynamic between changes over time in morbidity and in mortality, since fatal and nonfatal outcomes are generally correlated. However, postponement of morbidity by itself would improve health, while postponement of mortality by itself would increase ill-health. The dynamic interaction of morbidity trends and mortality trends was critical to accurate prediction of future health. If mortality was delayed the most, cumulative lifetime morbidity would grow; if morbidity was postponed more than mortality, cumulative lifetime morbidity would be likely to decrease. The prevalent aging paradigm of 1980, however, implicitly maintained that morbidity would continue to develop at a specific age, but that mortality could be postponed to an ever later age. Some even postulated no upper limit to human lifespan [2, 9, 10].

In retrospect, the inadequacy of the old paradigm is evident. At the time it began to be questioned, however, there were few data on trends in morbidity and trends in onset of morbidity could not be reliably estimated. Trends in mortality rates over many years, on the other hand, were readily ascertainable, reasonably accurate, and these rates were declining quite consistently over time. Gerontology was colloquially referred to as “the science of drawing downwardly sloping lines.” The concept of the plasticity (modifiability) of aging, where markers of aging could sometimes improve instead of inevitably decline, was not often discussed. More complicated models were needed, where the dynamic relationship between morbidity and mortality rates could be understood. In turn, we needed longitudinal population data on morbidity. We needed to be able to track the results of risk factors on both morbidity and mortality. Only as data became available could hypotheses of Compression of Morbidity be tested.

## 3. Science

Scholarly studies of Compression of Morbidity took several forms, the most definitive have involved (1) longitudinal study of morbidity in populations with differing risk factors, (2) population studies establishing decreases in population disability over long time periods, and (3) randomized controlled trials of health risk reduction in senior populations which showed decreased morbidity.

Other productive areas of study [11–16], noted but not elaborated here for reasons of space, include associations of health risk factors, morbidity, and increased medical care costs [11, 12]. Moreover, supercentenarians, over age 105, have proved to have had less lifetime cumulative morbidity than those dying at age 85 or 100 [13]. Programs based upon “active aging” concepts, most importantly exercise, have generally been found effective although many such studies were small, short-term, and not well controlled [14–16]. On the other hand, we are not aware of long-term studies of exercise which were not associated with reduction in disability [17]. Internationally, some populations studied over time have not experienced postponement of morbidity and some may have increased it, suggesting that while it is clearly possible to postpone morbidity under some circumstances [16], this result is not an inevitable one. 

## 4. Longitudinal Studies of Disability: Runners Club versus Community Controls

We began our two longitudinal studies of aging in 1984 and 1986 and results have been reported formally every few years [18–23]. These studies were designed to directly test the hypothesis of compression of morbidity by lifestyle choices. Here, we describe these studies informally, and readers wishing study details should refer to the referenced papers. In this paper, we present new data including mortality rate trends beyond the median age at death and analyze mortality data out to 24 years of study. We are now able to directly compare postponement of morbidity and mortality in the same cohorts, and postponement of disability is greater than that of mortality.

The “Runners Study” began in 1984 with recruitment of 538 senior runners and 423 age-matched (average age 58) controls. Runners were deliberately recruited from the “50 Plus Runner's Club,” for the most part jogged or ran over 2000 miles a year and were exercise enthusiasts. The control group was drawn randomly from the same community; about 25% of controls also ran recreationally, although they only averaged about 10% of the yearly distances logged by Runners Club members. Thus, the study allowed self-selection bias into the Runners cohort, conservatively included a very healthy control group. Analyses were focused in large part upon identifying, and if necessary adjusting for, selection biases [18–20]. 

The design was intended to achieve as great a difference as possible between groups in the independent variable of interest. In this case, we wanted a large difference between the exercise group and the controls in “vigorous exercise minutes per week.” We also wanted to create groups which were similar in educational attainment and income levels; lower levels of these socioeconomic factors are well known to be associated with poorer health and were potentially confounding variables for our study. Initial differences between groups in the exercise variable were tenfold, sufficient to dwarf possible confounders such as increased body mass index and cigarette smoking, which were rarely reported by either cases or controls. In analyses we also controlled for gender, ethnicity, physical injuries, family histories of arthritis, baseline X-rays for arthritis, chronic illnesses, whether they had ever run for exercise for a month or more, initial disability levels, and many other variables. Statistically adjusted data never differed significantly from raw data in any analyses. More complete discussion of these analytic issues may be found elsewhere [18–20].

**Figure 1:**
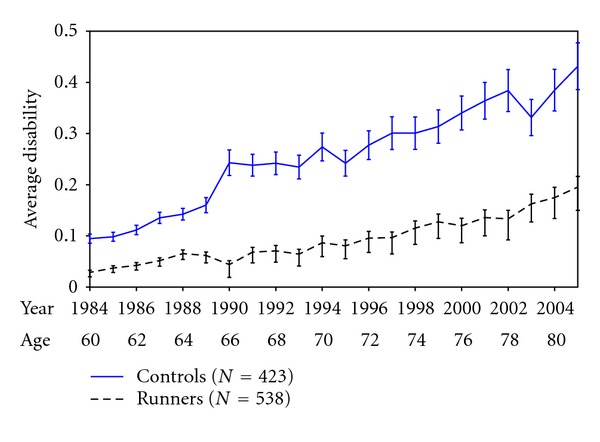
Average disability scores by age and calendar year, Runner's and community controls 1984–2005.

**Figure 2:**
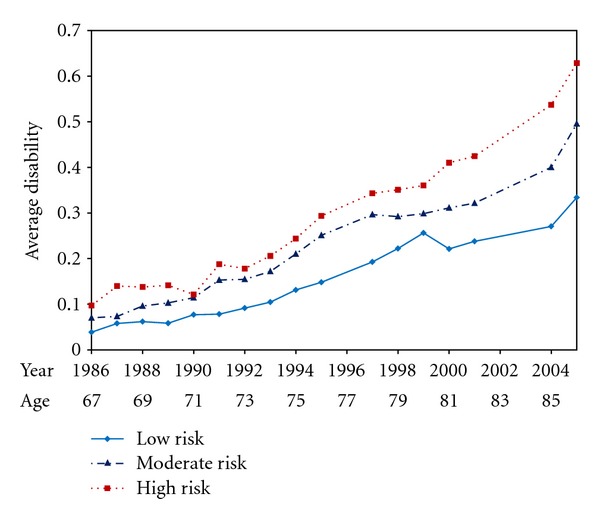
Average disability scores by age and calendar year, University of Pennsylvania Study 1986–2005.

**Figure 3:**
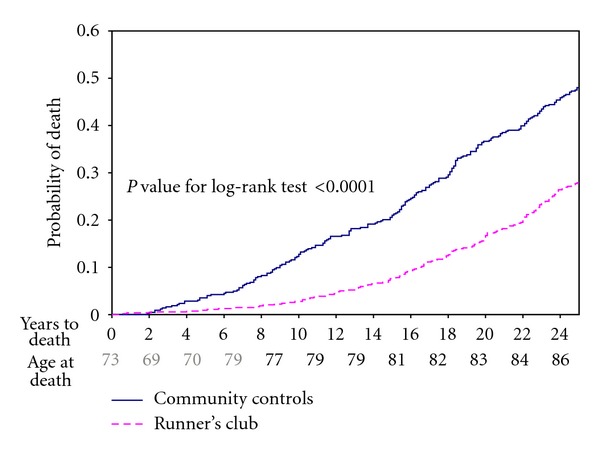
Kaplan-Meier analysis, Runner's and community controls 1984–2009.

**Figure 4:**
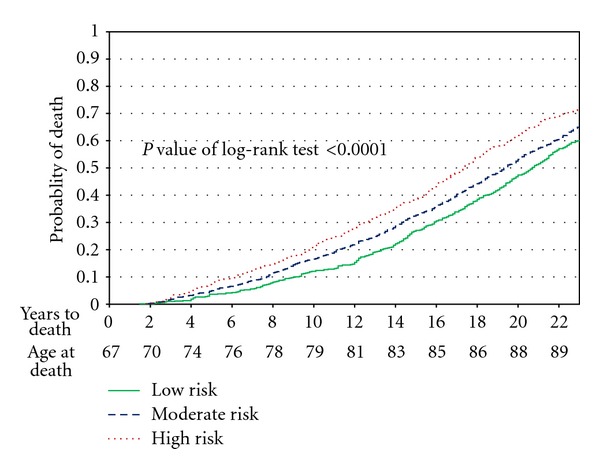
Kaplan-Meier analysis, University of Pennsylvania 1986–2009.

Our primary analyses have been longitudinal study of the two original cohorts established in 1984. We also analyzed the “ever-runners” versus the “never-runners” cohorts formed in 1984 in order to exclude a bias where those who ran but stopped because of some physical difficulty and ended up in the control group; results were similar to those when we used the original runners and control cohorts but even more striking. Primary endpoints were between cohort differences on the horizontal axis over time rather than cross-sectional differences on the vertical axis (Figures 1–4). We sought to determine how long, if at all, disability was postponed in the Runners cohort compared with Controls [21].

Figure 1 shows disability levels [3] and 95% confidence limits from 1984 to 2005, comparing the Runners with the Controls. The Runners had slightly less disability at study onset in 1984, believed due to their prior 10 years (on average) of vigorous exercise. Over the years through an average age of 80, the differences in disability between the runners cohort and controls grew steadily greater and regression lines continued to diverge (*P* < 0.001). The postponement of minimal (0.1 units) disability was 14 years over controls, and postponement of a higher disability level of 0.2 units was 16 years [3]. In other analyses, runners reported substantially less bodily pain and utilized substantially fewer medical resources [22]. In an X-ray subset, runners had a nonstatistically significant trend (4 versus 12) toward fewer knee replacements and totally destroyed (bone-on-bone) knee joints [19]. 

These findings were robust to statistical adjustments. We believe that developing cohorts with a large difference in the independent variable, exercise, materially strengthened these results. It was similar in design to choosing to study lung cancer incidence in 4-pack-a-day smokers versus nonsmokers; the differences in lung cancer incidence would be very large and study would not require very many subjects to reach statistical significance. Of interest, about a third of runners in both cohorts discontinued running over the years. Reasons for discontinuation were generally social: the dog died, the subject moved to another climate, and running got boring. Essentially no one stopped running because of pain or arthritis. Almost all who stopped running continued other vigorous exercise through swimming, bicycling, brisk walking, or other activities. Thus, this is a study of regular vigorous activity rather than solely of long-distance running.

## 5. Longitudinal Studies of Disability: Risk Factors of Inactivity, Obesity, and Smoking

In the health risks cohorts (University of Pennsylvania) study, we have followed 1741 University of Pennsylvania attendees in 1939 and 1940 who were studied again in the College Alumni Study in 1962, and annually by our group beginning in 1986 at an average age of about 70 years. We formed three cohorts using data obtained in the College Alumni Study when they were in mid-life with an average of 43 years old. We did this to lock subjects into study cohorts with their mid-life health habits well before the media or the public knew much about these health risks, a conservative approach. Also conservative was to score only three health risks, arguably the most important ones, and to use simple sums of binary variables to define cohorts.

The risk factors were current smoking, body mass index (BMI) 25 or higher (overweight), and absence of vigorous physical activity (inactivity), including jogging, brisk walking, and other activities which resulted in a sweat, all as measured at age 43. The risk factor score was based upon low risk (no risk factors), moderate risk (1 risk factor), and high risk (2 or 3 risk factors). Thus, the risk score used to define the three cohorts was a priori, arbitrary, and simple, and did not permit “data mining” of baseline scores of multiple variables to bias results. Some study power was probably lost through use of a simple index, but objectivity was increased and, as it turned out, there was plenty of statistical power. Study details may be found in previous reports [21–23].

The dependent variables were mortality and morbidity (disability), as measured in the Runners study, at yearly intervals. The plan here was to begin with cohorts about 10 years older than in the Runners study so as to study aging effects at higher ages, to use college classmates so that entry ages were clustered, and to select university alumni as a means to reduce confounding by poverty and other social disadvantage, as well as to maintain good follow-up rates and accurate reporting. Subjects were not aware of their membership in a particular cohort [23].

Figure 2 shows disability scores by age and by calendar year for the three cohorts from 1984 to 2005. The risk factor cohorts of low (0 risk factors), moderate, (1 risk factor), and high risk (2 or 3 risk factors) had initial scores in the postulated order, where the low risk cohort had less initial disability than the moderate risk cohort, which had less than the high risk cohort. Initial disability levels were all close to zero, however, and baseline effects were small. Disability levels and differences between cohorts increased monotonically over time. At last observation in 2005, high-risk subjects were about twice as disabled as low risk. Similar results obtained when we looked at cumulative disability, those living, those who had died, and men and women, and when we adjusted for covariates [23].

A disability score of 0.3 units (moderate disability) was postponed by 10 years in low-risk subjects compared with high risk. Since there were also differences in mortality, which was highest in high risk, noncompleters due to death were occurring particularly in the high-risk subjects with the very highest risks, acting against the primary findings. Attrition other than by death did not differ between cohorts. The relative contribution of each of the three risk factors was difficult to estimate because of autocorrelation of the risk factors [23]. 

## 6. Longitudinal Studies of Mortality: Runners versus Controls

In our study of runners and controls we have complete mortality data confirmed by the National Death Index from 1986 through 2009, a period of 25 years [18]. Two hundred and seven controls, out of 423 (48.9%) had died, compared with 164 out of 538 in the runners cohort (30.5%). Thus, we are able to compare differences between cohorts at the level where at least 30 percent had died in each cohort, but not at the median.

Figure 3 displays a life table comparison of the two cohorts. The runners had only 25% of the mortality rates of the control group over the first eight years, but there was subsequent convergence as subjects aged so that at year 25 the runners have 60 percent of the mortality rate of the Controls. Over the most recent five years the mortality curves are parallel or even closing slightly [18]. Median death for the controls is about 83 years of age; median age at death cannot yet be estimated for the exercising group but will be higher. 

There are differences in the morbidity and mortality outcome variables in that mortality is binary with a metric of years to death, while morbidity (disability) is considered as a continuous variable scored from zero to three, usually with a monotonically upward trend in the individual once nonzero disability has been noted. It is difficult to estimate these outcomes validly until most subjects have died in all cohorts and one can compare median values. In Figure 3, the postponement of mortality is about 7 years in the runners at last observation, but this difference seems likely to close during the age period of 83 to 93 years by which time most of the subjects in each cohort will have died. Postponement of morbidity (Figure 1) is 14 to 16 years. Spousal validation studies did not reveal questionnaire or interview responses of morbidity of either runners or controls to be biased in either direction [18–23]. 

## 7. Longitudinal Studies of Mortality: Health Risk Factors

The low risk (no risk factors), moderate risk (1 risk factor), and high risk (2 or 3 risk factors) cohorts had overall mortality of 60%, 65%, and 72%, respectively in 2009, so that all cohorts had passed the median death and all had at least reached the 60% mortality level.  Figure 4 shows Kaplan-Meier survival curves for these cohorts. Differences favoring the low-risk cohort tended to diverge increasingly from the high-risk cohort over the first 12 years and then to stabilize and to slightly converge subsequently. 

Postponement of mortality is seen more clearly here than in the Runners study since a greater fraction of subjects have died. We estimate this difference by measuring the horizontal difference between the high- and the low-risk groups at each decile mortality line which meets or crosses all three curves (0.1–0.6). At last observation, mortality postponement is about 3.5 years, and it has been three to four years throughout. The median death occurred about 3.7 years later in the low-risk group as compared with the High risk [21, 23]. Using linear regression analyses to smooth the curves, postponement of death is 3.3 years in low risk versus high risk, with moderate risk always intermediate. This postponement was 2.5 years in men and 4.0 years in women.

Our estimates of postponement of disability in the two studies thus range from 10 to 16 years, and postponement of death ranges from 3.3 to 7 years. These results are specific to these risk factor distributions and these patient cohorts. The data, replicated by these parallel studies and backed by the general literature, suggest that exercise improves health, that nonsmoking is a healthy habit, and that a normal body weight is good for you, both in terms of mortality and also in terms of cumulative lifetime disability and other life quality measures. They suggest that postponement of disability absent these risk factors is several-fold the postponement of mortality and thus that the onset of disability draws closer to the age at death, compressing morbidity between a 10- to 16-year later onset and only a 3.3- to 7-year postponed age at death [21].

Contrasting the two studies, effects are similar for both mortality and morbidity, but the differences are less in the health risk cohorts than in the runners. These results suggest that lack of exercise may be the most important risk factor of them all, particularly in nonsmokers. However, the Runners study had a very large difference in the independent variable, vigorous physical exercise, by design. The risk factor cohorts were derived from a more homogeneous population with lesser differences in the prevalence of risk factors between cohorts.

## 8. Population Studies of Morbidity

Another major effort to test the Compression hypothesis has involved population studies over time. In the United States, two premier population studies in the United States were begun shortly after the Compression of Morbidity hypothesis was raised. The National Long-Term Care Survey (NLTCS) [24, 25] studied Medicare eligible subjects 65 years old and older whether institutionalized or community-living (1982–2004) and the National Health Interview Study (NHIS) noninstitutionalized individuals over age 70 (1982–1999) [26]. Disability in the NLTCS declined 1.27% over the entire period and 2.1% in the last five years. NHIS had similar results, as did the next five studies as ranked by quality [27]. Mortality rates declined nationally about 1% per year over this period [28]. This documents the possibility of Compression of Morbidity on a national basis. It should be noted that not all studies have shown morbidity compression, particularly some European studies, and there has been speculation, and a little data, suggesting that the current obesity epidemic might reverse improvements of the prior two decades; the data presented here in the Risk Factor study could be considered to support this possibility. Unfortunately, the NLTCS had its last survey cycle in 2004, and more recent data from other sources cannot be directly compared to the NLTCS because of multiple changes in sampling designs and outcome variable definitions.

Population studies, because of their broad reach and policy implications, are of great value. Nevertheless, they are not particularly sensitive to change, and they generally offer little insight into the causes of the changes. National mortality and morbidity rates are influenced by economic cycles and offsetting trends in risks, as in rising population obesity and declining population cigarette smoking. They do not necessarily reflect a coherent population trend in health risks. It is not surprising that different countries have reported different trends, some of which differences do not appear to be attributable to methodology [29]. 

These studies provide proof-of-concept, in that the best studies since 1982 in the United States show convincing rates of Compression of Morbidity [30]. But, Compression of Morbidity, almost a necessity for progress in health improvement, clearly has not occurred in all populations and all subgroups [31].

## 9. Need and Demand Reduction

The Compression paradigm is central to health policy issues. If Compression of Morbidity occurs, it seems likely that the medical care burden would also go down. Costs of chronic diseases in senior citizens are a large driver of medical costs. Medical care costs are threatening the viability of the economy. Disability is a strong predictor of medical costs [32–34]. Health promotion programs which reduce senior health risks, as studied with large randomized trials, can reduce health risks and also reduce costs [11, 12, 32]. The policy formulation “reduction in need and demand for medical services,” suggests an approach to reducing medical care costs by reducing the illness burden and thus reducing the need for medical services [33, 34]. The “demand” side reflects a population tendency to desire the new and the expensive rather than the older and traditional. 

Medical need theoretically may be moderated by improved lifestyle choices as described here, as well as use of medical self-care, hospices, advance directives, and other low-cost interventions. The Centers for Medicare and Medicaid Services currently is studying the role of interventions directed at the twin goals of improving health and reducing costs, and preliminary results of a large randomized multiyear study of such interventions in Medicare populations should be available soon [35]. The “Compression of Morbidity” is an important theoretical approach to both health improvement and medical care cost reduction [36].

## 10. Trajectories of Morbidity: The Algebra of Health

Another promising research agenda involves study of the discrete trajectories which collectively describe overall health outcomes in a population. Many factors other than personal health risks affect both population mortality and morbidity, and a current challenge is to catalog the positive or negative effects of such factors upon mortality and morbidity. In addition to health risk reduction and its association with compression of morbidity, other potential factors include safer cars, super highways, seat belts, neighborhoods, ethnicity, prompt treatment of stroke, hypertension control, glucose control, cholesterol control, total joint replacement, HIV/AIDS, gang murder, and suicide before mid-life. 

Moreover, there are subsets of disease conditions that result in opposite trajectories, as with fatal and nonfatal heart attacks, fatal and non-fatal strokes, or resectable versus non-resectable cancer. For example, coronary artery disease has a trajectory of sudden mid-life death as the first symptom, a trajectory of sudden death with first symptom at an advanced age, a trajectory of multiple acute coronary events, and a trajectory of slow progression of chronic congestive heart failure. 

The effects of coronary artery disease on the national morbidity and mortality thus require descriptive information on the several trajectories, the incidence of each, and the algebraic sum of trajectories as the impact of the disease condition. Deconstruction of the possible trajectories of a disease is followed by reconstruction. Presently available data as to the direction and magnitude of effect of disease trajectories permits conjecture but not conclusion [21, 36, 37]. Future advances in health assessment and health policy will require detailed study of the different trajectories of the same disease.

The new paradigm of “Active Aging” or “Compression of morbidity” aspires to improve human health. There is proof-of-concept. Future population health improvement will require theory, science, and strategy and will involve multiple disciplines of inquiry.
